# Integrating reflex, feedback, and prediction: a tiered model of physiological regulation for contemporary medical education

**DOI:** 10.3389/fphys.2026.1817927

**Published:** 2026-08-20

**Authors:** Changiz Mohiyeddini, Suzan Kamel-ElSayed

**Affiliations:** 1 Michael Garron Hospital, Toronto East Health Network, Toronto, ON, Canada; 2 Department of Family and Community Medicine, Temerty Faculty of Medicine, University of Toronto, Toronto, ON, Canada; 3 Department of Foundational Medical Studies, Oakland University William Beaumont School of Medicine, Rochester, MI, United States

**Keywords:** allostasis, heart rate variability, homeostasis, medical education, neurovisceral integration, physiological regulation, predictive processing, psychophysiology

## Abstract

Medical education traditionally presents physiological regulation through homeostatic feedback loops—reactive mechanisms that correct deviations from set points. More recently, allostatic models have emphasized the brain’s anticipatory role in regulation. However, presenting these frameworks as competing paradigms creates conceptual confusion for learners. This paper proposes a unified, three-tiered model of physiological regulation that positions reflexive, homeostatic, and predictive mechanisms as complementary systems operating across different timescales. Tier 1 encompasses local reflexive responses providing millisecond-to-second protection. Tier 2 comprises systemic homeostatic feedback maintaining core variables over minutes to hours. Tier 3 involves brain-centered predictive regulation integrating cognition, emotion, and context to anticipate demands. Critically, these tiers interact hierarchically: reflexive mechanisms provide immediate protective boundaries, homeostatic systems coordinate whole-body stability, and predictive processes adjust regulatory set points in anticipation of future needs. In turn, reflexive and homeostatic mechanisms constrain predictive regulation within biological limits. This hierarchical framework provides medical educators with coherent scaffolding for teaching regulation and prepares students to understand how psychological and contextual factors influence health outcomes.

## 1 Introduction

Human physiology is a complex and multifaceted system comprising a multitude highly inter-related components (Modell et al., 2015; Silverthorn, 2006). Consequently, teaching physiology or physiological aspects of human psychology is a challenging endeavor, as a solid understanding of human physiology is one of the key requirements (Finnerty et al., 2010; Michael, 2007).

Undoubtedly, comprehending physiological regulation is fundamental to medical education (Carroll, 2017; Michael et al., 2017; Modell et al., 2015). Accordingly, how physiological regulation is taught significantly shapes future physician’s conceptualization of health and disease. Currently, the dominant approach presents physiological regulation through the concept of homeostasis, in which internal variables are maintained within functional ranges by integrated feedback and feedforward mechanisms (Cannon, 1929; Guyton and Hall, 2021). This framework has proven pedagogically effective: students can study discrete regulatory systems—such as blood pressure, temperature, and glucose—with clear mechanistic explanations and tangible examples (Modell et al., 2015; Silverthorn, 2006). However, contemporary neuroscience has established that regulation is not merely reactive but profoundly shaped by anticipation, context, and belief (Sterling and Eyer, 1988; Schulkin and Sterling, 2019). The concept of allostasis reframes the brain as a predictive organ that prepares for demand and pre-adjusts physiological systems accordingly. From this perspective, classical feedback loops represent only part of the regulatory architecture.

The pedagogical concern arises when homeostasis and allostasis are presented as unrelated or mutually exclusive frameworks, rather than as complementary aspects of physiological regulation (Ramsay and Woods, 2014; Carpenter, 2004). Students encounter homeostasis in preclinical physiology, then meet allostasis in behavioral medicine, often without guidance on how these models integrate (Kulasegaram et al., 2013; Finnerty et al., 2010). The result is conceptual fragmentation.

This paper proposes an integrated, holistic, and three-tiered model that positions different regulatory modes as complementary mechanisms (Ramsay and Woods, 2014; Sterling, 2012). Instead of replacing homeostasis with allostasis, we articulate how reflexive protection, homeostatic correction, and predictive anticipation integrate together and how understanding their interactions elucidates both physiology and pathophysiology.

## 2 The limits of linear models

### 2.1 The fragmentation problem

Traditional organ-system curricula present and teach regulation as independent loops (Finnerty et al., 2010; Kulasegaram et al., 2013; Michael, 2007). Students learn the baroreceptor reflex in cardiovascular physiology, glucose-insulin dynamics in endocrinology, and thermoregulation separately. Each loop is presented with mechanistic detail, yet connections between loops—and between physiological regulation and psychological processes—remain implicit (Borrell-Carrió et al., 2004).

This fragmented curriculum and teaching mode creates specific educational deficits (Kulasegaram et al., 2013). When a patient’s hypertension responds poorly to pharmacotherapy but improves with stress reduction, students lack a framework for understanding why (Esler, 2017; Bacon et al., 2004). When anxiety exacerbates irritable bowel syndrome, the connection between emotional state and gut motility appears mysterious rather than mechanistically explicable (Brosschot et al., 2018; Critchley and Harrison, 2013).

### 2.2 The false dichotomy

Compounding fragmentation is the tendency to present homeostasis and allostasis as opposing paradigms (Ramsay and Woods, 2014). This framing misrepresents both frameworks. Homeostatic feedback mechanisms remain essential—no amount of anticipatory regulation can substitute for immediate corrective responses when blood pH drops or hemorrhage threatens collapse. What allostasis contributes is recognition that feedback mechanisms operate within a broader regulatory context shaped by learning, expectation, and meaning (Sterling, 2012).

### 2.3 The set-point rigidity

Traditional presentations imply that set points are fixed values (Carpenter, 2004; Modell et al., 2015). This obscures their dynamic nature (Ramsay and Woods, 2014). Fever represents adaptive upward shift in the temperature set point. Circadian rhythms systematically vary cardiovascular and metabolic parameters. Chronic stress recalibrates cortisol dynamics. A model that treats physiological set points as fixed cannot easily account for context-dependent regulation; recognizing that higher-order brain processes can adjust defended levels better explains these observations.

### 2.4 The three-tiered model of regulation

**Figure 1:**
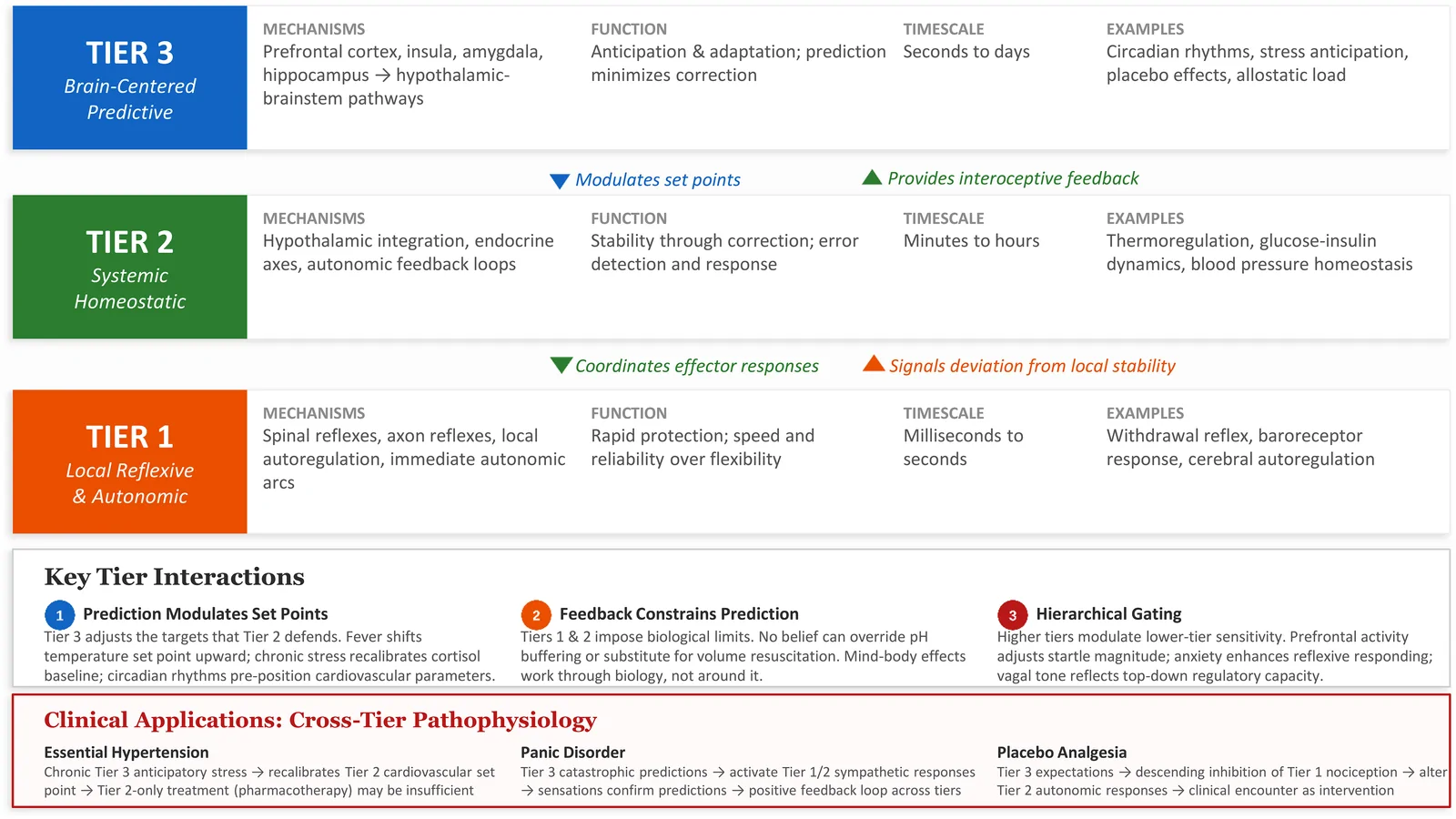
The three-tiered model of physiological regulation illustrating hierarchical organization and tier interactions. Tier 1 (orange) provides rapid, reflexive protection operating on millisecond-to-second timescales. Tier 2 (green) comprises classical homeostatic feedback maintaining core variables over minutes to hours. Tier 3 (blue) encompasses brain-centered predictive regulation integrating cognition, emotion, and context to anticipate demands. Bidirectional arrows indicate key interactions: predictive processes modulate homeostatic set points (downward), while homeostatic systems provide interoceptive feedback informing predictions (upward). The interaction panel summarizes three fundamental modes of cross-tier influence. Clinical examples demonstrate how dysfunction propagating across tiers produces recognizable pathology and implies multi-tiered treatment strategies.

We propose that physiological regulation operates holistically and coordinated through three complementary tiers, each characterized by distinct mechanisms, timescales, and adaptive functions (**Figure 1**; **Table 1**).

**Table 1:** Characteristics of the three tiers of physiological regulation.

Tier	Name	Mechanisms	Adaptive function	Timescale	Key principle	Examples
1	Local Reflexive & Autonomic	Spinal reflexes, axon reflexes, autoregulation, immediate autonomic arcs	Rapid protection	Milliseconds to seconds	Speed and reliability trump flexibility	Withdrawal reflex, baroreceptor response, cerebral autoregulation
2	Systemic Homeostatic	Hypothalamic integration, endocrine axes, autonomic feedback loops	Stability through correction	Minutes to hours	Error correction with tolerance for delay	Thermoregulation, glucose-insulin dynamics, fluid-electrolyte balance
3	Brain-Centered Predictive	Prefrontal-insular-limbic circuits, hypothalamic-brainstem pathways	Anticipation and adaptation	Seconds to days	Prediction minimizes correction	Circadian rhythms, stress anticipation, placebo effects, allostatic load

#### 2.4.1 Tier 1: local reflexive and autonomic regulation

The first tier encompasses rapid, largely hardwired responses that protect tissues from immediate threat. These mechanisms operate on milliseconds to seconds, often unnoticed by human consciousness (Dampney, 2016; Purves et al., 2018).

##### 2.4.1.1 Mechanisms

Tier 1 includes spinal reflexes (Pierrot-Deseilligny and Burke, 2012), axon reflexes mediating local vasodilation, autoregulatory responses maintaining organ perfusion (Willie et al., 2014), and immediate autonomic adjustments such as cardiac responses to sudden postural change (Dampney, 2016).

##### 2.4.1.2 Adaptive function

Rapid protection—minimizing immediate damage before slower systems respond. When a hand contacts a hot surface, withdrawal occurs before conscious pain perception (Purves et al., 2018). When blood pressure drops suddenly, baroreceptor-mediated heart rate increases begin within one cardiac cycle (Dampney, 2016).

##### 2.4.1.3 Key principle

In these mechanisms, speed and reliability take priority over flexibility.

These mechanisms are inflexible precisely because flexibility would introduce dangerous delays. The baroreflex is engaged during hemorrhage, dehydration, and postural change because each creates an immediate need to maintain arterial pressure and cerebral perfusion (Dampney, 2016; Willie et al., 2014).

##### 2.4.1.4 Evidence

Spinal reflex arcs demonstrate response latencies of 20–50 milliseconds (Pierrot-Deseilligny and Burke, 2012). Cerebral autoregulation maintains perfusion across wide pressure ranges through myogenic and metabolic mechanisms intrinsic to vasculature (Willie et al., 2014).

#### 2.4.2 Tier 2: systemic homeostatic feedback

The second tier comprises classical homeostatic mechanisms that detect deviations from target values, integrate information centrally, and activate effector responses to restore physiological equilibrium (Cannon, 1929; Guyton and Hall, 2021).

##### 2.4.2.1 Mechanisms

Tier 2 includes thermoregulatory responses (Morrison and Nakamura, 2019; Romanovsky, 2018), insulin and glucagon secretion maintaining glycemic stability (Röder et al., 2016), respiratory adjustments to altered blood gases, and fluid-electrolyte balance through renal and hormonal mechanisms (Guyton and Hall, 2021).

##### 2.4.2.2 Adaptive function

Stability through correction—maintaining core variables within ranges compatible with cellular function over minutes to hours.

##### 2.4.2.3 Key principle

Error correction with tolerance for delay which, unlike Tier 1, affords processing time because threats unfold more gradually. Core temperature can drift several degrees before cellular damage (Romanovsky, 2018); blood glucose can vary substantially before neurological deterioration (Röder et al., 2016).

##### 2.4.2.4 Evidence

Thermoregulation involves hypothalamic integration of thermoreceptor inputs with effector outputs including vasomotion, sweating, shivering, and behavioral responses (Morrison and Nakamura, 2019). Glucose homeostasis depends on pancreatic islet responses modulated by incretin hormones, autonomic inputs, and hepatic sensing (Röder et al., 2016).

#### 2.4.3 Tier 3: brain-centered predictive regulation

The third tier encompasses anticipatory, context-sensitive regulatory functions emphasized by allostatic theory (Sterling and Eyer, 1988; Schulkin and Sterling, 2019). Forebrain structures integrate interoceptive signals, environmental cues, memory, and expectations to predict demands and pre-adjust physiological systems (Critchley and Harrison, 2013; Thayer and Lane, 2000).

Importantly, characterizing Tier 3 as “brain-centered” does not imply that the brain functions as a central controller issuing commands to subordinate systems. Rather, the brain’s predictive computations emerge from the organism’s developmental and experiential history—they are products of embodied interaction with the environment, not programs written by an external agent. Moreover, anticipatory properties are not exclusive to the brain. Peripheral tissues also exhibit forms of adaptive memory: skeletal muscle, for instance, retains epigenetic signatures of prior mechanical loading through DNA hypomethylation and myonuclear accretion, enabling enhanced hypertrophic responses upon retraining even after prolonged detraining (Seaborne et al., 2018; Wen et al., 2021). Such peripheral anticipatory adaptation operates without requiring centrally mediated prediction. What distinguishes Tier 3 in the present model is not a claim that all anticipation resides in the brain, but rather that the forebrain uniquely integrates contextual, emotional, and cognitive information to generate regulatory adjustments that span multiple organ systems simultaneously.

##### 2.4.3.1 Mechanistic specificity

Tier 3 involves prefrontal and insular cortical processing of context, hippocampal contributions to contextual memory, amygdalar evaluation of threat, and hypothalamic-brainstem pathways translating these computations into autonomic, endocrine, and immune outputs (Critchley and Harrison, 2013; Garfinkel et al., 2015; Thayer and Lane, 2000).

Although Tier 3 processes are distributed across multiple neural systems, key pathways are increasingly well characterized. Stress-related predictive regulation operates through infralimbic and prelimbic prefrontal projections to the central nucleus of the amygdala and bed nucleus of the stria terminalis (BST), which in turn modulate paraventricular nucleus (PVN) output to initiate HPA axis activation (Ulrich-Lai and Herman, 2009; Herman et al., 2005). This cascade produces measurable cortisol elevation within 15–30 minutes of stressor onset, with peak concentrations at approximately 30–45 minutes (Dickerson and Kemeny, 2004). Catecholaminergic projections from the locus coeruleus to prefrontal and limbic structures provide rapid arousal signaling that shapes the initial appraisal context within seconds (Aston-Jones and Cohen, 2005). Vagal afferent pathways convey visceral state information to the nucleus tractus solitarius and onward to insular cortex, closing the interoceptive loop through which Tier 3 processes monitor and adjust Tier 2 parameters (Critchley and Harrison, 2013). These pathways operate through identifiable neurotransmitter systems — glucocorticoids, norepinephrine, acetylcholine, and corticotropin-releasing hormone — rather than through abstract psychological influence. What distinguishes Tier 3 mechanistically is not immateriality but complexity: multiple converging inputs are integrated to generate context-sensitive regulatory outputs that vary with learning, memory, and expectation.

##### 2.4.3.2 Adaptive function

Anticipation and adaptation—optimizing physiological readiness for predicted challenges and minimizing energetic costs of reactive correction (Sterling, 2012; Schulkin and Sterling, 2019). For instance, if the brain predicts increased metabolic demand, pre-adjusting cardiovascular parameters reduces the regulatory burden on Tier 2 systems.

##### 2.4.3.3 Key principle

Prediction minimizes the need for correction. Circadian pre-awakening rises in cortisol prepare the body for sleep-to-activity transition (Rao and Androulakis, 2019). Pre-competition increases in heart rate prepare athletes for exertion before it begins (Thayer et al., 2009).

##### 2.4.3.4 Evidence

Functional neuroimaging demonstrates that anticipation of pain, reward, or social evaluation activates prefrontal, insular, and limbic regions projecting to autonomic centers (Wager et al., 2004). Heart rate variability predicts individual differences in emotional regulation capacity and cardiovascular health (Thayer et al., 2009).

### 2.5 Tier interactions: the core of the model

The framework’s pedagogical power lies in its articulation of tier interactions. Understanding how reflexive protection, homeostatic correction, and predictive anticipation integrate together and how understanding their interactions elucidates both physiology and pathophysiology.

### 2.6 The core concept: prediction modulates set points

The traditional view treats homeostatic set points as fixed biological constants. The system detects deviation, activates correction, and restores the target value. Our model challenges this assumption: Tier 3 (brain-centered, predictive) processes can adjust the target itself that Tier 2 defends. The thermostat isn’t fixed; the brain can turn the dial up or down based on anticipated needs.

This resolves an apparent paradox: how can homeostasis, which emphasizes stability, coexist with allostasis, which emphasizes change? Homeostatic mechanisms continue to detect deviations and initiate corrective responses, but the defended level around which regulation occurs can be adjusted by higher-order brain processes. The system is simultaneously stable (in mechanism) and flexible (in target).

### 2.7 Fever as paradigm

#### 2.7.1 What happens mechanistically

##### 2.7.1.1 Immune trigger

Bacterial infection releases pathogen-associated molecular patterns (PAMPs). Immune cells respond by releasing pyrogenic cytokines—primarily IL-1β, IL-6, and TNF-α.

##### 2.7.1.2 Hypothalamic signal

These cytokines reach the hypothalamus (either crossing the blood-brain barrier at circumventricular organs or signaling via vagal afferents). In the preoptic area, they induce prostaglandin E2 (PGE_2_) synthesis.

##### 2.7.1.3 Set-point shift

PGE_2_ acts on thermosensitive neurons, effectively telling the hypothalamus: “The target temperature is now 39 °C, not 37 °C.” (Evans et al., 2015; Romanovsky, 2018).

##### 2.7.1.4 Homeostatic machinery activates—but toward the NEW target

The body now perceives 37 °C as “too cold.” Full Tier 2 corrective responses engage:

- Shivering generates heat
- Vasoconstriction conserves heat
- Behavioral responses (seeking blankets, curling up) reduce heat loss
- The patient feels cold and acts accordingly

##### 2.7.1.5 The new set point is defended

Once core temperature reaches 39 °C, these responses cease. The system is now in “equilibrium”—just at a higher temperature.

#### 2.7.2 Pedagogical relevance

Students often think fever means “thermoregulation has broken,” when in reality thermoregulation is working perfectly — it’s just defending a different target (Romanovsky, 2018). The Tier 2 machinery (sensors, integrators, effectors) functions normally. What changed was a Tier 3 intervention: the immune-brain axis predicted that elevated temperature would enhance immune function — which it does: fever increases lymphocyte proliferation, antibody production, and pathogen killing (Evans et al., 2015) — and adjusted the target accordingly.

#### 2.7.3 The allostatic interpretation

Fever is predictive preparation. The brain anticipates the metabolic demands of fighting infection and pre-adjusts physiology to optimize immune performance. The “cost” (increased metabolic rate, discomfort) is tolerated because it serves survival.

### 2.8 Circadian modulation

#### 2.8.1 What happens mechanistically

##### 2.8.1.1 The master clock

The suprachiasmatic nucleus (SCN) in the anterior hypothalamus contains ~20,000 neurons with intrinsic ~24-hour oscillations driven by transcription-translation feedback loops (*CLOCK/BMAL1* → *PER/CRY* → inhibition → cycle repeats) (Dibner et al., 2010).

##### 2.8.1.2 Light entrainment

Retinal ganglion cells (using melanopsin) project directly to the SCN, synchronizing the internal clock to the external light-dark cycle.

##### 2.8.1.3 Systemic coordination

The SCN doesn’t directly control peripheral organs. Instead, it orchestrates timing signals via autonomic projections, hormonal signals (especially cortisol and melatonin), body temperature rhythms, and behavioral cycles (Clow et al., 2010; Scheiermann et al., 2018).

##### 2.8.1.4 Peripheral clocks obey

Nearly every cell in the body contains the same clock genes. These peripheral oscillators are synchronized by SCN signals, creating system-wide temporal coordination.

### 2.9 Why this is Tier 3 (predictive) rather than Tier 2 (reactive)

These variations (**Table 2**) aren’t responses to detected deviations; however they’re anticipatory adjustments. Blood pressure rises before the individual wakes up, not in response to standing. Cortisol peaks before activity begins. The SCN is essentially “predicting” the daily cycle and pre-positioning physiological systems.

**Table 2:** Circadian variation in key physiological parameters and their adaptive functions.

Parameter	Pattern	Adaptive function
Cortisol	Peaks ~30 min after waking, lowest at midnight	Prepares for daytime metabolic demands
Blood pressure	Rises before waking, dips during sleep	Cardiovascular readiness for activity
Heart rate	Higher during day, lower at night	Matches cardiac output to activity level
Body temperature	Peaks late afternoon, troughs early morning	Optimizes enzyme function during active period
Insulin sensitivity	Higher in morning, lower at night	Matches glucose handling to feeding times
Immune function	Inflammatory responses peak at night	Tissue repair during rest

#### 2.9.1 The allostatic interpretation

Circadian rhythms minimize the corrective burden on Tier 2 systems. If blood pressure is already elevated when the individual stands, the baroreceptors have less deviation to correct. If cortisol is already mobilizing glucose when the individual wakes up, glycemic regulation faces less demand. Prediction reduces the cost of correction.

#### 2.9.2 Clinical relevance

Circadian disruption (shift work, jet lag, social jet lag) uncouples the SCN from peripheral clocks. This forces Tier 2 systems to handle larger corrections because the predictive pre-positioning is misaligned. The result: increased cardiovascular risk, metabolic dysfunction, impaired immune function as documented in shift workers (Baron and Reid, 2014; Scheer et al., 2009).

### 2.10 Stress-induced recalibration (allostatic load)

#### 2.10.1 What happens mechanistically

##### 2.10.1.1 Acute stress response (adaptive)

A stressor activates the HPA axis and sympathetic nervous system. Cortisol rises, heart rate increases, inflammatory responses mobilize. This is normal, adaptive Tier 3 activation preparing for challenge.

##### 2.10.1.2 Recovery (normal)

When the stressor ends, negative feedback (cortisol suppressing CRH and ACTH) restores baseline. Parasympathetic activity rebounds. The system returns to pre-stress operating parameters.

##### 2.10.1.3 Chronic stress (maladaptive)

When stressors are persistent, unpredictable, or perceived as uncontrollable, the system doesn’t fully recover between activations. Several things go wrong:

- Receptor downregulation: Chronically elevated cortisol causes glucocorticoid receptors in the hippocampus and hypothalamus to downregulate. This weakens negative feedback, so cortisol remains elevated longer (McEwen, 1998).
- Set-point drift: The HPA axis “resets” to a higher baseline. What was once “stressed” becomes the new “normal” (McEwen and Stellar, 1993).
- Inflammatory priming: Chronic cortisol exposure can paradoxically increase inflammatory sensitivity. Pro-inflammatory gene expression increases; anti-inflammatory regulation weakens (Marsland et al., 2017; Slavich and Irwin, 2014).
- Autonomic imbalance: Sympathetic tone remains elevated; vagal tone decreases. Resting heart rate increases; heart rate variability decreases (Thayer et al., 2012).

##### 2.10.1.4 The new “normal” is pathological

The system now defends these altered parameters as if they were appropriate. The regulatory systems continue to function, yet they stabilize the body around pathological baselines that were established during chronic stress (McEwen, 1998, McEwen, 2008).

#### 2.10.2 McEwen’s allostatic load concept

McEwen (1998) and McEwen and Stellar, 1993) distinguished between the following (**Table 3**):

- Allostasis: Adaptive, predictive adjustment to meet anticipated demands (beneficial)
- Allostatic load: Cumulative wear-and-tear from chronic allostatic activation (harmful)
- Allostatic overload: When the load exceeds the system’s capacity, producing overt pathology (McEwen, 2008)

**Table 3:** Biomarkers of allostatic load across regulatory systems (adapted from McEwen, 1998; McEwen and Stellar, 1993; Picard et al., 2014).

System	Marker	Change
HPA axis	Cortisol awakening response	Blunted or exaggerated
HPA axis	Diurnal cortisol slope	Flattened
Cardiovascular	Resting blood pressure	Elevated
Cardiovascular	Heart rate variability	Reduced
Metabolic	Waist-hip ratio	Increased
Metabolic	HDL cholesterol	Decreased
Inflammatory	C-reactive protein, IL-6	Elevated
Cellular	Telomere length	Shortened

### 2.11 Why this is a Tier 3 → Tier 2 interaction

The origin of the recalibration is Tier 3: psychological perception of chronic threat/demand drives sustained predictive activation (**Table 4**). But the consequence is altered Tier 2 operating parameters: the homeostatic machinery now defends elevated blood pressure, altered cortisol rhythms, and primed inflammatory responses as if these were appropriate baselines.

**Table 4:** Set-point modulation: examples of Tier 3 → Tier 2 interaction.

Example	Tier 3 signal	Effect on Tier 2	Adaptive/maladaptive	Clinical implication
Fever	Immune cytokines signal infection	Temperature set point shifts upward	Adaptive (enhances immune function)	Don’t always suppress fever; it serves function
Circadian rhythm	SCN predicts daily activity cycle	Cardiovascular, metabolic, endocrine parameters pre-adjust	Adaptive (reduces corrective burden)	Circadian disruption increases disease risk
Chronic stress	Persistent threat perception	HPA, cardiovascular, inflammatory baselines recalibrate	Maladaptive (allostatic load)	Must address psychological drivers, not just physiological targets

#### 2.11.1 Clinical relevance

This explains why “treating the numbers” often fails. Clinicians can pharmacologically lower blood pressure (Tier 2 intervention), but if chronic anticipatory stress (Tier 3) continues driving set-point elevation, the intervention is fighting the underlying regulatory signal (Esler, 2017). The model suggests that effective treatment may require addressing both tiers — combining pharmacotherapy with stress reduction, cognitive reframing, or contextual change (Bacon et al., 2004).

#### 2.11.2 Feedback constrains prediction

Tier 1 and Tier 2 mechanisms impose hard limits on predictive regulation. The brain’s anticipatory computations must operate within biological constraints.

#### 2.11.3 Non-negotiable parameters

Arterial pH must remain between approximately 7.35 and 7.45; departures produce rapid cellular dysfunction regardless of psychological state (Ramsay and Woods, 2014). No placebo effect can substitute for bicarbonate buffering when metabolic acidosis threatens.

#### 2.11.4 The boundary conditions of belief

Placebo effects show that expectations can influence pain perception, autonomic responses, and inflammatory activity (Benedetti, 2014; Wager and Atlas, 2015). However, these effects depend on the body’s existing biological systems—for example, placebo analgesia works through the endogenous opioid system. Expectations cannot replace biology: they cannot regenerate neurons or eliminate cancer. Understanding these boundaries helps prevent both therapeutic nihilism and unrealistic magical thinking.

#### 2.11.5 Hierarchical gating

Higher tiers modulate sensitivity and responsiveness of lower tiers.

#### 2.11.6 Cortical modulation of startle

The acoustic startle reflex, a Tier 1 brainstem-mediated response, is modulated by prefrontal activity. Anticipation reduces response magnitude; anxiety enhances it (Grillon, 2002). This gating allows contextual information in Tier 3 to adjust Tier 1 sensitivity (Thayer et al., 2009, 2012).

#### 2.11.7 Autonomic tuning

Prefrontal-vagal pathways allow Tier 3 processes to adjust autonomic balance. High heart rate variability reflects robust prefrontal capacity to modulate autonomic outflow; reduced variability suggests diminished top-down regulatory capacity (Thayer et al., 2009, Thayer et al., 2012).

**Figure 1** provides a visual summary of the three-tiered model, illustrating the hierarchical organization of regulatory mechanisms, their distinct timescales, and the bidirectional interactions through which predictive, homeostatic, and reflexive processes continuously influence one another.

### 2.12 Clinical correlates

The three-tiered framework illuminates clinical phenomena that appear puzzling when viewed through a single-tier lens.

### 2.13 Essential hypertension

Primary hypertension often resists explanation in purely Tier 2 terms (Esler, 2017). The tiered model suggests examining Tier 3 contributions: chronic anticipatory cardiovascular activation driven by perceived stress may recalibrate the operating range Tier 2 defends. Treatment addressing only Tier 2 may prove less effective than interventions also targeting Tier 3, because the latter addresses the predictive signals driving set-point elevation (Bacon et al., 2004).

### 2.14 Panic disorder

Panic attacks demonstrate how Tier 3 predictions activate Tier 1 and Tier 2 responses without actual threat. Misinterpretation of benign interoceptive signals as cardiac pathology triggers sympathetic activation (Critchley and Harrison, 2013), producing sensations that confirm the catastrophic interpretation (Khalsa et al., 2018). Treatment with interoceptive exposure addresses this cross-tier dynamic by recalibrating Tier 3 predictions about bodily sensations (Craske et al., 2014).

### 2.15 Placebo analgesia

Expectations of pain relief activate descending inhibitory pathways modulating spinal nociceptive processing (Tier 1) and alter autonomic responses (Tier 2) through identifiable neurobiological mechanisms (Benedetti, 2014; Wager and Atlas, 2015). Understanding placebo as a Tier 3 regulatory phenomenon positions the clinical encounter itself — clinician communication, patient expectations, meaning attributed to symptoms — as biologically consequential (Borrell-Carrió et al., 2004).

### 2.16 Pedagogical implementation

#### 2.16.1 Organizing existing content

The three-tiered model provides medical educators with a coherent strategy for integrating content that current organ-system curricula fragment (Kulasegaram et al., 2013; Silverthorn, 2006). For any organ system, educators can restructure content around the three tiers without discarding existing material.

Cardiovascular example:

- Tier 1: Local autoregulation, immediate baroreceptor responses
- Tier 2: Sustained blood pressure homeostasis, fluid-volume regulation
- Tier 3: Circadian cardiovascular variation, stress-induced hypertension, white-coat effect

This reorganization makes explicit the connections between physiological and psychological contributors to cardiovascular disease that traditional curricula leave implicit. Importantly, no existing content is lost. The baroreceptor reflex and fluid-volume regulation remain central. What changes is the addition of a contextual layer (Tier 3) that explains phenomena students will encounter clinically but currently lack the framework to understand mechanistically: why blood pressure readings differ between clinic and home, why chronic stress elevates cardiovascular risk, and why shift workers develop hypertension (Esler, 2017; Smolensky et al., 2017).

The same three-tier mapping applies to endocrine, respiratory, gastrointestinal, and immune systems, providing a unifying organizational logic across the curriculum. For example, in endocrinology: Tier 1 encompasses rapid insulin secretion responses; Tier 2 comprises glucose-insulin feedback dynamics; and Tier 3 addresses circadian variation in insulin sensitivity, stress-related metabolic dysregulation, and the role of anticipatory cephalic-phase responses in glycemic control. This cross-system applicability is a key pedagogical advantage — students learn one reasoning framework and apply it repeatedly rather than learning disconnected models for each system.

#### 2.16.2 Case-based integration

The application phase of team-based learning is designed to require students to integrate concepts and trace pathophysiology across regulatory tiers. Consider the following case:

A 45-year-old executive presents with poorly controlled hypertension despite pharmacologic treatment. His home blood pressure readings are consistently lower than those recorded in the clinic. He reports significant occupational stress.

Using the three-tier model of regulation, explain how reflexive, homeostatic, and predictive mechanisms may contribute to his presentation. How might this framework inform a more comprehensive treatment approach?

Students must recognize white-coat hypertension as Tier 3 anticipatory activation; consider whether chronic stress has recalibrated Tier 2 cardiovascular set points; determine whether Tier 2-targeted pharmacotherapy alone can succeed when Tier 3 processes drive dysregulation; and propose multi-tiered interventions combining medication with stress reduction, cognitive restructuring, or modification of environmental conditions generating chronic anticipation (Esler, 2017; Bacon et al., 2004).

This approach teaches students to ask not just “what is the deviation?” but “which tier is driving it?” — a shift from single-mechanism reasoning to integrative physiological thinking. The pedagogical value lies in requiring students to move beyond identifying a dysregulated variable toward diagnosing the regulatory level at which dysfunction originates and matching intervention accordingly.

Crucially, these cases need not be invented from scratch. Existing clinical vignettes used in physiology and pathophysiology courses can be adapted by adding contextual and psychological information and prompting students to analyze the contribution of each tier. This lowers the implementation barrier for educators working within established curricula.

#### 2.16.3 Reframing the set point

The model supports reframing set-point instruction. Rather than presenting set points as fixed constants, educators can use tiered examples to demonstrate their dynamic nature (Carpenter, 2004; Modell et al., 2015).

Three worked examples, already familiar from preclinical physiology, illustrate the principle: circadian processes shift cardiovascular targets predictively, preparing the body for anticipated demands before they arise (Clow et al., 2010; Smolensky et al., 2017); fever represents adaptive set-point elevation driven by immune-brain signaling, in which Tier 2 machinery defends an intentionally elevated target (Evans et al., 2015; Romanovsky, 2018); and chronic stress maladaptively recalibrates regulatory targets through sustained Tier 3 activation, producing allostatic load (McEwen, 1998).

This reconceptualization has direct clinical relevance. It helps students understand why treating symptoms — the Tier 2 deviation from a recalibrated target — without addressing the predictive signals driving recalibration (Tier 3) may yield incomplete clinical results. A patient whose blood pressure is pharmacologically normalized but whose chronic work stress continues will likely require escalating doses or additional agents, not because the medication has failed but because the regulatory signal driving set-point elevation remains active (Esler, 2017).

Teaching set points as dynamic, contextually modulated targets rather than fixed biological constants represents a fundamental shift in how students conceptualize both normal physiology and pathophysiology — and prepares them for the clinical reality that effective treatment often requires identifying and addressing the tier at which dysregulation originates.

## 3 Discussion

The three-tiered model presented here offers a conceptual framework that addresses longstanding pedagogical challenges in teaching physiological regulation. Rather than presenting homeostasis and allostasis as unrelated or mutually exclusive frameworks — a false dichotomy that fragments understanding — the model positions reflexive, homeostatic, and predictive mechanisms as complementary systems that evolved to solve different adaptive problems across different timescales.

### 3.1 Theoretical implications

The model’s core contribution lies in articulating tier interactions. The recognition that Tier 3 predictive processes can modulate Tier 2 homeostatic set points resolves an apparent paradox that has troubled the field: how can homeostasis (implying stability) coexist with allostasis (implying adaptive change)? The answer is that the homeostatic machinery remains stable while the targets it defends are dynamically adjustable. The thermostat works the same way whether set to 37 °C or 39 °C; what changes is the dial setting, not the mechanism.

This reframing has important consequences for how we conceptualize disease. Many chronic conditions as essential hypertension, metabolic syndrome, functional gastrointestinal disorders, chronic pain syndromes resist explanation as simple failures of homeostatic feedback. The tiered model suggests an alternative: these conditions may represent maladaptive recalibration of Tier 2 parameters driven by persistent Tier 3 predictive signals. The homeostatic machinery functions correctly; it simply defends inappropriate targets established through chronic stress, learned threat associations, or maladaptive beliefs about bodily states.

Equally important is the model’s articulation of constraints. Recognizing that Tier 1 and Tier 2 mechanisms impose biological limits on Tier 3 predictive processes prevents both therapeutic nihilism and magical thinking. The brain’s predictions cannot override arterial pH buffering, restore blood volume after hemorrhage, or regenerate infarcted tissue. Understanding these boundaries clarifies what mind-body interventions can and cannot accomplish. They work through biology, engaging endogenous opioid, autonomic, and inflammatory pathways, not around it.

### 3.2 Clinical implications

The tiered framework carries direct implications for clinical reasoning and treatment planning. When regulatory dysfunction involves multiple tiers, single-tier interventions may prove insufficient. As illustrated in the essential hypertension example (see Clinical Correlates), chronic anticipatory stress operating through Tier 3 can continuously recalibrate cardiovascular set points, rendering Tier 2-targeted pharmacotherapy alone inadequate (Esler, 2017; Bacon et al., 2004). This principle extends beyond hypertension. In functional gastrointestinal disorders, persistent anticipatory threat signaling (Tier 3) sustains visceral hypersensitivity and altered motility patterns that symptom-focused pharmacotherapy may fail to resolve (Brosschot et al., 2018). In chronic pain syndromes, maladaptive predictive models about bodily threat maintain central sensitization despite peripheral tissue healing — a Tier 3 process driving what appears to be Tier 1/2 dysfunction (Wager and Atlas, 2015). In each case, effective treatment requires identifying and addressing the tier at which dysregulation originates rather than defaulting to the tier at which symptoms manifest.

This multi-tiered perspective also illuminates why the clinical encounter itself is therapeutically consequential. Clinician communication, patient expectations, and the meaning patients attribute to their symptoms all operate through Tier 3 mechanisms. Placebo analgesia demonstrates this principle: expectations of relief activate descending inhibitory pathways, modulate autonomic responses, and alter inflammatory signaling through identifiable neurobiological mechanisms (Benedetti, 2014; Wager and Atlas, 2015). This is not an artifact to be controlled away but a demonstration of predictive regulation in action. A clinician who understands this recognizes that how they communicate diagnosis and prognosis directly participates in physiological regulation — positioning the therapeutic relationship as biologically consequential, not merely psychologically comforting (Borrell-Carrió et al., 2004).

The model further suggests that certain treatment failures may reflect tier mismatch. Interventions targeting Tier 2 (pharmacological correction of physiological parameters) may prove inadequate when pathology originates in Tier 3 (maladaptive predictions, learned threat associations, chronic anticipatory activation). Conversely, psychological interventions targeting Tier 3 cannot substitute for Tier 1 and Tier 2 interventions when immediate physiological correction is required — no amount of cognitive restructuring will stabilize a patient in diabetic ketoacidosis. Matching intervention to the tier driving dysfunction becomes a core clinical reasoning skill, one that the tiered framework makes explicitly teachable.

### 3.3 Tier interactions in health and resilience

The clinical applications presented thus far emphasize dysregulation — conditions in which tier interactions produce or sustain pathology. However, the tiered framework equally characterizes healthy regulation, and articulating what adaptive tier coupling looks like is essential for a balanced pedagogical model.

Healthy tier interactions can be characterized by three properties. First, appropriate set-point flexibility: Tier 3 predictive processes adjust Tier 2 parameters in proportion to actual contextual demands, and these adjustments reverse efficiently when demands subside. The cortisol awakening response illustrates this principle — a robust anticipatory rise followed by systematic decline across the day reflects well-calibrated Tier 3 modulation of Tier 2 endocrine function (Clow et al., 2010). Flattened or exaggerated cortisol slopes, by contrast, suggest degraded predictive calibration. Second, efficient recovery: following acute Tier 3 activation, parasympathetic rebound restores autonomic balance rapidly. High resting heart rate variability indexes this recovery capacity, reflecting robust prefrontal modulation of autonomic outflow (Thayer et al., 2009, Thayer et al., 2012). Third, accurate interoception: when Tier 3 predictive models are informed by faithful representation of Tier 2 states, regulatory adjustments are more precise and less prone to the catastrophic misinterpretation seen in conditions such as panic disorder (Garfinkel et al., 2015; Khalsa et al., 2018).

This characterization raises a question with direct clinical and educational relevance: can Tier 3 processes be trained to enhance regulatory function? Emerging evidence suggests they can. Mindfulness-based interventions, which cultivate non-reactive awareness of interoceptive signals, have been associated with changes in inflammatory markers and immune parameters consistent with improved Tier 3→Tier 2 regulation (Black and Slavich, 2016). Cognitive reappraisal — deliberately reframing the meaning of stressors — engages prefrontal circuits that modulate amygdalar and autonomic reactivity, effectively recalibrating the Tier 3 predictions that drive Tier 2 activation (Wager et al., 2004; Thayer and Lane, 2000). Heart rate variability biofeedback targets the Tier 3→Tier 2 interface directly, training individuals to enhance vagal tone through resonance frequency breathing (Lehrer and Gevirtz, 2014). These interventions are not merely psychological in nature; they operate through identifiable neural and autonomic pathways that the tiered model makes explicit.

Exercise-induced adaptation offers a particularly instructive example of positive tier coupling. Repeated physical loading produces systematic improvements in Tier 2 parameters—increased cardiac stroke volume, enhanced mitochondrial density, improved insulin sensitivity—that reflect not merely homeostatic correction but progressive recalibration of regulatory capacity. The phenomenon of overcompensation, in which physiological systems recover to levels exceeding pre-exercise baselines following appropriately dosed training loads, illustrates how repeated perturbation can enhance rather than degrade regulatory function—the adaptive counterpart to allostatic overload. Crucially, these adaptations involve regulatory memory distributed across multiple biological levels. At the cellular level, skeletal muscle retains epigenetic memory of prior hypertrophic loading through sustained DNA hypomethylation at genes involved in muscle growth, even after muscle mass has returned to baseline during detraining (Seaborne et al., 2018; Wen et al., 2021). This retained epigenetic signature enables larger and faster hypertrophic responses upon retraining, demonstrating that complex adaptive systems possess memory and learn from prior encounters with demand—properties that cannot be fully captured by classical feedback models. For students, exercise adaptation thus provides a concrete, clinically relevant example of how tier interactions can drive health rather than disease, and of how regulatory memory operates at tissue and cellular levels alongside the brain-centered predictive processes emphasized in Tier 3.

For the student, this perspective reframes prevention and wellness — not as the absence of pathology but as the active maintenance of adaptive tier coupling. A patient with high heart rate variability, flexible cortisol dynamics, and accurate interoceptive awareness is not simply “not sick” but is demonstrating robust multi-tiered regulatory coordination. Teaching students to recognize and promote these markers of regulatory health complements the pathology-focused reasoning that dominates traditional curricula.

### 3.4 Relationship to existing frameworks

The three-tiered model builds upon and integrates several established theoretical frameworks. Sterling and Eyer (1988) allostasis concept provides the foundation for Tier 3, emphasizing brain-centered predictive regulation. Sterling (2020) subsequently developed this perspective into a comprehensive account of health as efficient predictive regulation rather than the maintenance of fixed parameters — a view fully consistent with the tiered model’s treatment of set points as dynamically adjustable targets. McEwen (1998) elaboration of allostatic load explains how chronic Tier 3 activation produces cumulative physiological costs. Thayer and Lane (2000) neurovisceral integration model specifies the neural architecture—prefrontal-vagal pathways—through which Tier 3 processes modulate Tier 2 autonomic parameters. The model synthesizes these contributions within a hierarchical structure that preserves the validity of classical homeostatic concepts while incorporating predictive and contextual dimensions.

The framework also resonates with emerging perspectives in computational neuroscience, particularly predictive processing and active inference theories (Friston, 2010; Seth and Critchley, 2012). These accounts propose that the brain continuously generates predictions about expected sensory inputs—including interoceptive signals from the body—and acts to minimize prediction error. Within this framework, Tier 3 regulation represents the brain’s attempt to maintain predicted physiological states, with Tier 2 homeostatic mechanisms serving as the effector systems that implement these predictions. While a full integration with predictive processing accounts exceeds the present scope, the compatibility suggests productive theoretical convergence. Recent advances in interoceptive inference have further specified how the brain constructs predictive models of visceral states and how mismatches between predicted and actual interoceptive signals may drive both autonomic dysregulation and subjective symptoms (Barrett and Simmons, 2015; Owens et al., 2018; Quigley et al., 2021). These accounts map directly onto the tiered framework: interoceptive predictions are Tier 3 processes, the visceral states being predicted are Tier 2 parameters, and prediction errors represent the signals through which tiers communicate.

### 3.5 Relationship to complex adaptive systems and network physiology

The tiered model, as presented, employs a functional hierarchy to organize regulatory mechanisms for pedagogical purposes. This framing necessarily simplifies dynamics that, in the living organism, exhibit the properties of complex adaptive systems: nonlinearity, self-organization, dynamic stability, emergent coordination, and context-dependent synchronization across organ networks (Balagué et al., 2020; Ivanov, 2021). A full account of physiological regulation must ultimately engage with these properties, and the tiered framework should be understood as a pedagogical scaffold rather than a literal description of regulatory architecture.

The emerging field of network physiology offers a complementary perspective that addresses several limitations of hierarchical models. Where the tiered framework asks which functional mode of regulation—reflexive, homeostatic, or predictive—is driving a given physiological outcome, network physiology asks how the coordination and coupling dynamics among organ systems give rise to integrated physiological states (Balagué et al., 2020; Ivanov, 2021). In this view, health is characterized not by the state of individual regulatory parameters but by the topology and temporal dynamics of inter-organ network interactions—the strength, directionality, and flexibility of coupling between cardiovascular, respiratory, neural, and musculoskeletal systems. Disease and maladaptation reflect disruptions in network coordination rather than failures within isolated feedback loops.

This perspective highlights phenomena that the tiered model, in its current form, does not fully address. Anticipatory regulation is not confined to brain-centered processes; peripheral organs and tissues demonstrate anticipatory properties through local adaptive mechanisms. Skeletal muscle exhibits overcompensation following appropriately dosed training loads and retains epigenetic memory of prior adaptation through DNA hypomethylation (Seaborne et al., 2018; Wen et al., 2021), enabling enhanced future responses without requiring centrally mediated prediction. Cardiorespiratory coupling during exercise reflects emergent coordination among multiple organ systems that cannot be reduced to a single tier’s control (Balagué et al., 2020). These observations suggest that the organism’s regulatory intelligence is distributed across levels of biological organization rather than concentrated in a central controller.

The tiered model and network physiology are not mutually exclusive. The tiers identify functional modes of regulation—speed-reliability tradeoffs, error correction dynamics, and predictive optimization—that provide entry points for understanding what the network is doing. Network physiology provides the tools for understanding how these functional modes are coordinated through dynamic inter-organ coupling, synchronization, and self-organization. For medical education, the tiered model offers the conceptual vocabulary that students need before they can engage productively with the mathematical and dynamical complexity that network physiology entails. A student who understands that chronic stress can recalibrate cardiovascular set points through Tier 3 processes is better prepared to interpret network physiology data showing altered cardiorespiratory coupling under chronic stress than a student who has encountered neither framework. The tiered model is thus intended as a bridge—from organ-system reductionism toward integrative, systems-level physiological thinking—rather than a final destination.

### 3.6 Limitations

Several limitations warrant acknowledgment. First, the boundaries between tiers are heuristic rather than absolute. Neuroanatomical substrates overlap considerably — the hypothalamus participates in all three tiers, and prefrontal influences extend to brainstem reflexes through hierarchical gating. The tiers represent functional modes of regulation rather than anatomically discrete systems, and some regulatory processes may not map cleanly onto a single tier. More broadly, the tiered framework employs a functional hierarchy that necessarily simplifies the nonlinear, self-organizing, and emergent properties characteristic of complex adaptive systems (Balagué et al., 2020). Physiological regulation in the intact organism involves dynamic network interactions among organs and tissues—including reciprocal coupling, synchronization, and context-dependent reorganization—that exceed what a three-tier hierarchy can fully represent.

Second, the evidence base for Tier 3 modulation of specific Tier 2 parameters varies substantially. Strong evidence supports cognitive-emotional influences on autonomic function (Thayer et al., 2012), inflammatory markers (Marsland et al., 2017), and pain processing (Wager and Atlas, 2015). Evidence for direct cognitive influence on more tightly controlled parameters — arterial pH, plasma osmolarity, core body temperature outside fever — remains limited (Ramsay and Woods, 2014). The model should not be interpreted as claiming that all physiological variables are equally accessible to top-down modulation; rather, it proposes a framework for understanding which parameters are more or less amenable to predictive influence and why.

Third, individual differences in tier interactions remain poorly characterized. Genetic polymorphisms affecting glucocorticoid receptor sensitivity and catecholamine metabolism (e.g., *COMT*) likely moderate how effectively Tier 3 processes influence Tier 2 parameters (Federenko et al., 2006; Stein et al., 2008). Developmental history, including early adversity and attachment patterns, shapes the calibration of stress-responsive systems in ways that may alter tier interactions across the lifespan (Gunnar and Quevedo, 2007). Interoceptive accuracy — the ability to accurately perceive internal bodily signals — varies substantially across individuals and may determine how tightly Tier 3 predictions and Tier 2 states are coupled (Garfinkel et al., 2015; Khalsa et al., 2018). A student learning this model should understand that tier interactions operate probabilistically and variably, not deterministically and uniformly.

Fourth, the clinical applications presented — hypertension, panic disorder, placebo analgesia, functional gastrointestinal disorders — represent conditions where tier interactions are relatively well-established empirically. Extending the model to other conditions requires validation rather than assumption. Not every disease involves Tier 3 dysfunction, and over-attribution of illness to psychological factors risks both clinical error and patient harm (Sharpe and Greco, 2019).

Fifth, the model focuses primarily on dysregulation and pathology. Less attention has been given to positive adaptation, resilience, and the conditions under which tier interactions promote health rather than disease. Understanding why some individuals maintain healthy tier interactions despite chronic stress — the phenomenon of stress resilience — requires further theoretical development.

### 3.7 Future directions

Several research directions emerge from this framework. Longitudinal studies should examine how interventions targeting specific tiers alter biomarkers associated with other tiers over time. For instance, does cognitive-behavioral therapy for chronic stress produce measurable recalibration of inflammatory set points? Does mindfulness training alter the cortisol awakening response (Black and Slavich, 2016)? What is the time course of such recalibration, and how durable are the effects? These questions require designs that measure Tier 3 processes (psychological variables, neural activation patterns) alongside Tier 2 parameters (autonomic, endocrine, immune markers) over extended follow-up periods.

Mechanistic studies using concurrent neuroimaging and peripheral physiological measurement could map the neural pathways through which predictive computations translate into homeostatic parameter shifts. Simultaneous fMRI and autonomic recording during anticipatory tasks, for instance, could clarify how prefrontal-insular activity relates to cardiovascular adjustment in real time. Such studies would move the field beyond correlational associations between psychological states and physiological parameters toward a mechanistic account of tier interactions.

The role of interoception deserves particular attention as a potential moderator. If Tier 3 predictions depend on accurate representation of Tier 2 states, individuals with impaired interoceptive accuracy may show disrupted tier coupling (Garfinkel et al., 2015). This has clinical implications: conditions characterized by interoceptive dysfunction — alexithymia, depersonalization, some presentations of autism spectrum disorder — may exhibit distinctive patterns of tier interaction (Khalsa et al., 2018). Interventions enhancing interoceptive accuracy might, in principle, improve regulatory coordination across tiers.

Developmentally, understanding how tier interactions are established and calibrated during critical periods could inform prevention strategies. Early adversity appears to recalibrate stress-responsive systems in ways that persist into adulthood (Gunnar and Quevedo, 2007); specifying these effects in tiered terms could clarify intervention targets. Does early intervention prevent maladaptive Tier 3 → Tier 2 coupling, or can recalibration occur throughout the lifespan?

Educationally, controlled studies should evaluate whether teaching physiology through the tiered framework improves clinical reasoning compared to traditional approaches (Silverthorn, 2006). Outcome measures might include students’ ability to generate integrative differential diagnoses, their recognition of psychophysiological contributors to disease, and their communication with patients about mind-body interactions. Development of validated assessment tools for “tiered thinking” would support such research. Qualitative studies exploring how students conceptualize regulation before and after tiered instruction could identify persistent misconceptions and guide curricular refinement.

Technologically, the tiered model could be operationalized through simulation platforms and AI-driven educational tools. Virtual patient scenarios could be designed to require cross-tier reasoning — presenting cases where Tier 2 parameters shift in response to contextually manipulated Tier 3 inputs such as simulated stress, circadian disruption, or placebo conditions. Adaptive learning systems could track whether students default to single-tier explanations and provide targeted feedback promoting integrative reasoning. Such technologies would both implement the tiered framework pedagogically and generate data for educational research on the development of tiered thinking.

Implementation research should examine practical barriers to adopting the tiered model within existing curricula. Medical education remains largely organized by organ system or discipline; the tiered model is inherently trans-disciplinary. Integration may require dedicated sessions, case-based learning spanning traditional boundaries, or restructured assessment that rewards synthesis across domains. Faculty development will be essential, as many educators were trained within the fragmented paradigm the model seeks to transcend.

Finally, translational research should develop and test multi-tiered treatment protocols. If tier interactions shape disease trajectories as proposed, treatment algorithms should systematically address multiple tiers rather than defaulting to single-tier (usually Tier 2) interventions. What combinations of pharmacotherapy, behavioral intervention, circadian optimization, and attention to the therapeutic relationship produce optimal outcomes for specific conditions? Comparative effectiveness research designed around tiered principles could inform precision medicine approaches that match intervention strategy to the tier(s) driving individual patients’ dysfunction.

## 4 Conclusion

The three-tiered model of physiological regulation—encompassing local reflexive mechanisms, systemic homeostatic feedback, and brain-centered predictive control—offers medical educators a coherent framework for integrating content that current curricula fragment. By positioning these regulatory modes as complementary rather than mutually exclusive, the model resolves the apparent tension between classical physiology and contemporary neuroscience. Students no longer need to choose between homeostasis and allostasis; they can understand both as components of a hierarchical system in which rapid protection, error correction, and anticipatory optimization work together to maintain the conditions for life.

The model’s pedagogical power lies in its explicit articulation of tier interactions. Predictive processes modulate homeostatic set points, explaining phenomena from fever to circadian variation to stress-induced hypertension. Homeostatic and reflexive mechanisms constrain predictive control, establishing the biological boundaries within which mind-body interactions operate. Hierarchical gating allows context processed at higher tiers to adjust the sensitivity and responsiveness of lower tiers. These interactions, once made explicit, illuminate clinical phenomena that remain puzzling when viewed through a single-tier lens: why panic attacks mimic cardiac pathology, why chronic stress elevates cardiovascular risk, why the clinical encounter itself can accelerate or impede recovery (**Table 5**).

**Table 5:** Sources of individual variation in Tier interactions.

**Source**	**Category**	**Effect on tier interactions**	**Clinical relevance**	**Key references**
*COMT* Val158Met polymorphism	Genetic	Modulates prefrontal catecholamine availability, affecting Tier 3 regulatory capacity	Patients with Met/Met variant may show stronger Tier 3→Tier 2 coupling under stress	Stein et al., 2008; Mier et al., 2010
*FKBP5* polymorphisms	Genetic	Alters glucocorticoid receptor sensitivity, affecting HPA negative feedback efficiency	May slow Tier 2 recovery from Tier 3-driven stress activation	Binder, 2009
5-HTTLPR short allele	Genetic	Modifies serotonergic modulation of amygdala reactivity, influencing Tier 3 threat appraisal	Associated with heightened Tier 3 activation to ambiguous stimuli	Stein et al., 2008
Early adversity	Developmental	Recalibrates HPA axis set points and autonomic baseline through sustained Tier 3→Tier 2 activation during critical periods	Adult patients may show altered cortisol rhythms and reduced HRV reflecting developmental reprogramming	Gunnar and Quevedo, 2007; McEwen, 1998
Attachment history	Developmental	Shapes social regulation of Tier 3 processes; insecure attachment may diminish safety-signal effectiveness	Therapeutic alliance may be less physiologically regulating for patients with insecure attachment	Thayer and Lane, 2000
Interoceptive accuracy	Acquired	Determines fidelity of Tier 2→Tier 3 feedback; poor accuracy may decouple predictive models from physiological states	Relevant in panic disorder, alexithymia, and functional somatic syndromes	Garfinkel et al., 2015; Khalsa et al., 2018
Learned expectations	Acquired	Shapes Tier 3 predictive models through conditioning and prior experience	Placebo/nocebo responses vary with expectation history; prior treatment failure may alter Tier 3 predictions	Benedetti, 2014; Wager and Atlas, 2015
Sleep deprivation	State	Impairs prefrontal function, reducing Tier 3 regulatory capacity over Tier 1/2	Acutely sleep-deprived patients may show diminished top-down regulation and exaggerated autonomic reactivity	Thayer et al., 2012
Current stress load	State	Cumulative allostatic load shifts Tier 2 baselines and depletes Tier 3 regulatory resources	Patients under high concurrent stress may respond differently to identical interventions	McEwen, 1998; Picard et al., 2014

The tiered model predicts that individual differences in genetics, developmental history, acquired capacities, and current physiological state moderate how tiers interact. **Table 5** summarizes key sources of variation organized by category. For the clinician-in-training, the pedagogical implication is that two patients presenting with the same Tier 2 dysregulation — for example, elevated resting blood pressure — may differ substantially in the Tier 3 processes driving that dysregulation and in the biological substrates mediating tier coupling. Recognizing these sources of variation moves students beyond one-size-fits-all reasoning toward the individualized, multi-level clinical thinking the tiered framework is designed to cultivate.

For the clinician, the model suggests that effective treatment often requires addressing multiple tiers. Pharmacological correction of physiological parameters (Tier 2) may prove insufficient when maladaptive predictions (Tier 3) continuously recalibrate the targets being defended. Conversely, psychological interventions cannot substitute for immediate physiological correction when Tier 1 or Tier 2 threats demand rapid response. Matching intervention to the tier(s) driving dysfunction becomes a clinical reasoning skill that the tiered framework can help cultivate.

For the educator, the model provides scaffolding for curriculum design. Any organ system can be taught through its three tiers: the local protective mechanisms, the systemic corrective feedback, and the predictive contextual modulation. Case-based learning can require students to trace pathophysiology across tiers, recognizing how dysfunction at one level propagates to others. Assessment can reward integrative reasoning that spans traditional disciplinary boundaries.

Significant work remains. The evidence base for tier interactions varies across physiological systems and requires continued empirical investigation. Individual differences in tier responsiveness—shaped by genetics, development, and interoceptive capacity—demand further characterization. Educational research should evaluate whether tiered instruction improves clinical reasoning and patient care outcomes. Implementation requires addressing practical barriers within existing curricular structures.

Despite these limitations, the tiered model represents a step toward a more unified and clinically relevant approach to teaching physiological regulation. By preparing students to think in terms of hierarchical, interactive, context-sensitive control, we equip them for a medicine that is both mechanistically rigorous and attentive to the psychological and social dimensions of health. The human organism is neither a simple machine executing feedback loops nor a purely psychological entity immune to biological constraint; it is a multi-tiered regulatory system in which reflex, feedback, and prediction continuously collaborate. Teaching this integrative perspective may prove essential for addressing the chronic, stress-related, and functional conditions that increasingly dominate modern medical practice—and for cultivating clinicians capable of seeing patients not as collections of organ systems but as integrated, embodied, meaning-making beings navigating an uncertain world.

## Data Availability

The original contributions presented in the study are included in the article/supplementary material. Further inquiries can be directed to the corresponding author.
